# Tris(phenyl 2-pyridyl ketone oxime-κ^2^ *N*,*N*′)cadmium(II) dinitrate

**DOI:** 10.1107/S1600536809017073

**Published:** 2009-05-14

**Authors:** Juan Yan, Guang-Xiang Liu

**Affiliations:** a Anhui Key Laboratory of Functional Coordination Compounds, School of Chemistry and Chemical Engineering, Anqing Normal University, Anqing 246003, People’s Republic of China

## Abstract

The Cd atom in the title compound, [Cd(C_12_H_10_N_2_O)_3_](NO_3_)_2_, adopts a distorted octa­hedral geometry, being ligated by six N atoms from three different phenyl-2-pyridyl ketone oxime ligands. In the crystal structure, inter­molecular O—H⋯O and C—H⋯O hydrogen bonds link the mol­ecules into a chain structure propagating along [100]. The chains are further linked into a three-dimensional supra­molecular structure *via* van der Waals forces.

## Related literature

For related structures, see: Korpi *et al.* (2005 ▶); Pearse *et al.* (1989 ▶); Afrati *et al.* (2005 ▶); Stamatatos *et al.* (2006 ▶). For related literature on 2-pyridyl-substituted oximes, see: Papatrianta­fyllopoulou *et al.* (2007 ▶).
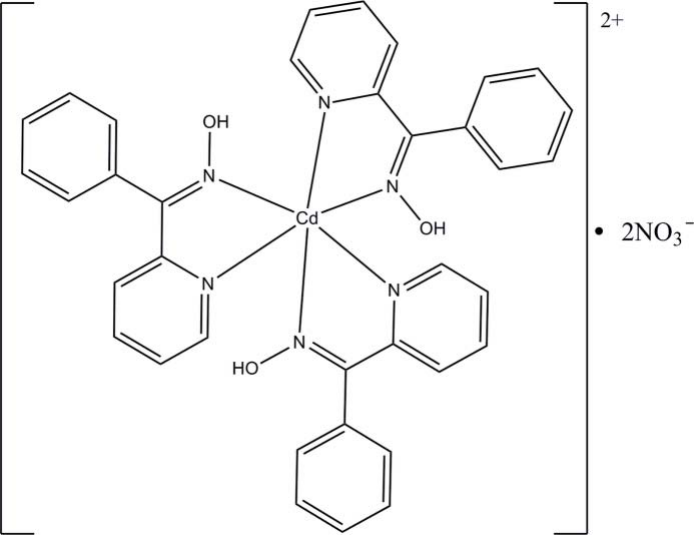


## Experimental

### 

#### Crystal data

[Cd(C_12_H_10_N_2_O)_3_](NO_3_)_2_
*M* *_r_* = 831.08
Triclinic, 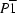

*a* = 10.618 (3) Å
*b* = 11.687 (4) Å
*c* = 15.279 (5) Å
α = 101.263 (4)°
β = 100.166 (4)°
γ = 101.807 (4)°
*V* = 1773.3 (10) Å^3^
*Z* = 2
Mo *K*α radiation
μ = 0.68 mm^−1^
*T* = 293 K
0.24 × 0.22 × 0.18 mm

#### Data collection

Bruker SMART Apex CCD area-detector diffractometer
Absorption correction: multi-scan (*SADABS*; Bruker, 2000 ▶) *T*_min_ = 0.853, *T*_max_ = 0.887
13329 measured reflections
6526 independent reflections
4387 reflections with *I* > 2σ(*I*)
*R*_int_ = 0.041

#### Refinement

*R*[*F* ^2^ > 2σ(*F* ^2^)] = 0.046
*wR*(*F* ^2^) = 0.077
*S* = 1.08
6526 reflections
490 parameters
H-atom parameters constrained
Δρ_max_ = 0.45 e Å^−3^
Δρ_min_ = −0.54 e Å^−3^

### 

Data collection: *SMART* (Bruker, 2000 ▶); cell refinement: *SAINT* (Bruker, 2000 ▶); data reduction: *SAINT*; program(s) used to solve structure: *SHELXS97* (Sheldrick, 2008 ▶); program(s) used to refine structure: *SHELXL97* (Sheldrick, 2008 ▶); molecular graphics: *SHELXTL* (Sheldrick, 2008 ▶); software used to prepare material for publication: *SHELXTL*.

## Supplementary Material

Crystal structure: contains datablocks I, New_Global_Publ_Block. DOI: 10.1107/S1600536809017073/at2779sup1.cif

Structure factors: contains datablocks I. DOI: 10.1107/S1600536809017073/at2779Isup2.hkl

Additional supplementary materials:  crystallographic information; 3D view; checkCIF report

## Figures and Tables

**Table 1:** Hydrogen-bond geometry (Å, °)

*D*—H⋯*A*	*D*—H	H⋯*A*	*D*⋯*A*	*D*—H⋯*A*
C32—H32⋯O8	0.93	2.48	3.355 (6)	157
C27—H27⋯O9^i^	0.93	2.58	3.191 (6)	123
C14—H14⋯O5^ii^	0.93	2.49	3.261 (6)	141
C4—H4⋯O3^iii^	0.93	2.42	3.285 (5)	155
C3—H3*A*⋯O2^iii^	0.93	2.56	3.461 (5)	164
O3—H3⋯O6	0.82	2.34	2.892 (4)	126
O3—H3⋯O4	0.82	2.00	2.820 (5)	176
O2—H2⋯O8	0.82	2.04	2.800 (5)	154
O2—H2⋯O7	0.82	2.21	2.939 (6)	149
O1—H1⋯O6	0.82	1.81	2.585 (4)	158

Symmetry codes: (i) 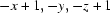; (ii) 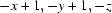; (iii) 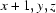.

**Fig. 1.**
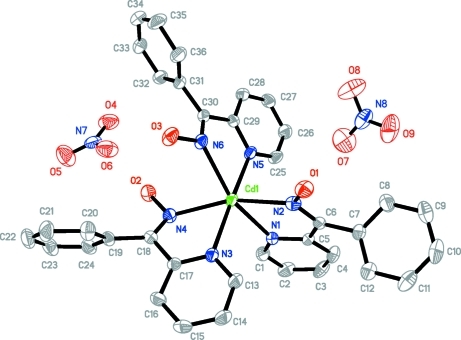
The molecular structure of the title compound, with atom labels and 30% probability displacement ellipsoids. H atoms have been omitted for clarity.

**Fig. 2.**
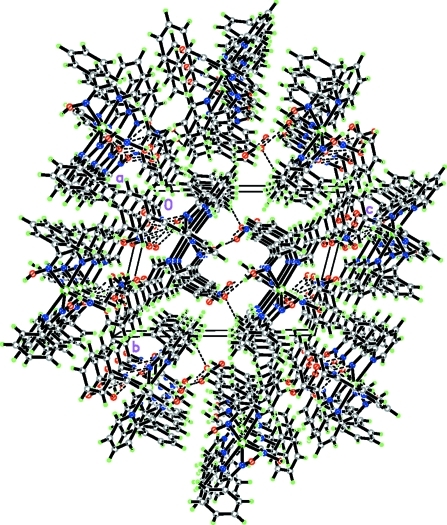
A packing diagram of the title compound. Hydrogen bonds are shown as dashed lines.

## supplementary crystallographic information

### Comment

Pyridine-2-carbaldehyde oxime ligands usually bind to metals in a bidentate fashion, either chelating one metal center or bridging two metals. Their complexes find application in diverse areas such as functional supramolecular design, magnetic materials and catalysis (Korpi *et al.*, 2005; Pearse *et al.*, 1989; Afrati *et al.*, 2005; Stamatatos *et al.*, 2006). The title compound is a new cadmium complex from the reaction of Cd(NO_3_)_2_.4H_2_O with phenyl-2-pyridyl ketone oxime (ppo). The compound consists of three N,N-chelating ligands and two nitrate anion. The three ppo ligands are coordinated to nickel to form three five-membered CdC_2_N_2_ rings.

The central cadmium atom adopts a distorted octahedral geometry (Fig. 1), which are ligated by six N atoms from three different phenyl-2-pyridyl ketone oxime ligands. The bond distances Cd—N, are in the expected ranges of 2.320 (3)–2.402 (3) Å, and the coordination angles around Ni atom are in the range 68.00 (11)–172.80 (12)°. In the crystal structure, intermolecular C—H···O hydrogen bonds link the molecules into one-dimensional chain structure, which are further linker into three-dimensional supramolecular structure *via* van der Waals forces.

### Experimental

A colourless solution of phenyl-2-pyridyl ketone oxime (0.394 g, 2.00 mmol) in MeOH (10 ml) was slowly added to a solution of Cd(NO_3_)_2_.4H_2_O (0.308 g, 1.00 mmol) in MeOH (10 ml) and the resulting colourless solution was stirred for 1 h at room temperature. A small quantity of undissolved material was removed by filtration. The filtrate was allowed to stand undisturbed in a closed flask for a period of 7–8 d. Colourless block crystals appeared which were collected by filtration, washed with cold MeOH (1 ml) and ice-cold Et_2_O (2 ml), and dried in air [yield: 70%].

### Refinement

H atoms were positioned geometrically, with C—H = 0.93 Å and O—H = 0.82 Å, and constrained to ride on their parent atoms, with *U*_iso_(H) = xU_eq_(C), where *x* = 1.5 for hydroxyl H and *x* = 1.2 for aromatic H atoms. The deepest hole is located 1.01Å from atom Cd1.

### Crystal data

**Table:**

[Cd(C_12_H_10_N_2_O)_3_](NO_3_)_2_	*Z* = 2
*M**_r_* = 831.08	*F*(000) = 844
Triclinic, *P*1	*D*_x_ = 1.556 Mg m^−^^3^
Hall symbol: -P 1	Mo *K*α radiation, λ = 0.71073 Å
*a* = 10.618 (3) Å	Cell parameters from 3787 reflections
*b* = 11.687 (4) Å	θ = 2.4–24.4°
*c* = 15.279 (5) Å	µ = 0.68 mm^−^^1^
α = 101.263 (4)°	*T* = 293 K
β = 100.166 (4)°	Block, colourless
γ = 101.807 (4)°	0.24 × 0.22 × 0.18 mm
*V* = 1773.3 (10) Å^3^	

### Data collection

**Table:**

Bruker SMART Apex CCD area-detector diffractometer	6526 independent reflections
Radiation source: sealed tube	4387 reflections with *I* > 2σ(*I*)
graphite	*R*_int_ = 0.041
phi and ω scans	θ_max_ = 25.5°, θ_min_ = 1.4°
Absorption correction: multi-scan (*SADABS*; Bruker, 2000)	*h* = −12→12
*T*_min_ = 0.853, *T*_max_ = 0.887	*k* = −13→14
13329 measured reflections	*l* = −18→17

### Refinement

**Table:**

Refinement on *F*^2^	Primary atom site location: structure-invariant direct methods
Least-squares matrix: full	Secondary atom site location: difference Fourier map
*R*[*F*^2^ > 2σ(*F*^2^)] = 0.046	Hydrogen site location: inferred from neighbouring sites
*wR*(*F*^2^) = 0.077	H-atom parameters constrained
*S* = 1.08	*w* = 1/[σ^2^(*F*_o_^2^) + (0.010*P*)^2^ + 0.9209*P*] where *P* = (*F*_o_^2^ + 2*F*_c_^2^)/3
6526 reflections	(Δ/σ)_max_ < 0.001
490 parameters	Δρ_max_ = 0.45 e Å^−^^3^
0 restraints	Δρ_min_ = −0.54 e Å^−^^3^

### Special details

**Table:**

Geometry. All e.s.d.'s (except the e.s.d. in the dihedral angle between two l.s. planes) are estimated using the full covariance matrix. The cell e.s.d.'s are taken into account individually in the estimation of e.s.d.'s in distances, angles and torsion angles; correlations between e.s.d.'s in cell parameters are only used when they are defined by crystal symmetry. An approximate (isotropic) treatment of cell e.s.d.'s is used for estimating e.s.d.'s involving l.s. planes.
Refinement. Refinement of *F*^2^ against ALL reflections. The weighted *R*-factor *wR* and goodness of fit *S* are based on *F*^2^, conventional *R*-factors *R* are based on *F*, with *F* set to zero for negative *F*^2^. The threshold expression of *F*^2^ > σ(*F*^2^) is used only for calculating *R*-factors(gt) *etc*. and is not relevant to the choice of reflections for refinement. *R*-factors based on *F*^2^ are statistically about twice as large as those based on *F*, and *R*- factors based on ALL data will be even larger.

### Fractional atomic coordinates and isotropic or equivalent isotropic displacement parameters (Å^2^)

**Table:**

	*x*	*y*	*z*	*U*_iso_*/*U*_eq_	
Cd1	0.39932 (3)	0.31776 (3)	0.24172 (2)	0.04876 (12)	
N1	0.6295 (3)	0.3685 (3)	0.2886 (2)	0.0444 (9)	
N2	0.5000 (3)	0.2396 (3)	0.1219 (2)	0.0427 (8)	
N3	0.4239 (3)	0.4973 (3)	0.1957 (2)	0.0421 (8)	
N4	0.3093 (3)	0.4563 (3)	0.3321 (2)	0.0421 (8)	
N5	0.3952 (3)	0.1499 (3)	0.3045 (2)	0.0426 (9)	
N6	0.1810 (3)	0.1985 (3)	0.2194 (2)	0.0424 (8)	
N7	0.1902 (3)	0.3451 (4)	0.0246 (3)	0.0670 (12)	
N8	0.3849 (5)	0.2736 (4)	0.5251 (3)	0.0751 (13)	
O1	0.4373 (3)	0.1901 (3)	0.02985 (19)	0.0568 (8)	
H1	0.3608	0.1964	0.0215	0.085*	
O2	0.2339 (3)	0.4255 (3)	0.39257 (19)	0.0524 (8)	
H2	0.2688	0.3854	0.4232	0.079*	
O3	0.0693 (3)	0.2320 (3)	0.1822 (2)	0.0615 (8)	
H3	0.0886	0.2782	0.1498	0.092*	
O4	0.1362 (3)	0.3998 (3)	0.0776 (2)	0.0938 (12)	
O5	0.2140 (4)	0.3763 (4)	−0.0428 (3)	0.1087 (14)	
O6	0.2210 (3)	0.2537 (4)	0.0439 (3)	0.0946 (12)	
O7	0.4502 (5)	0.3435 (4)	0.4894 (3)	0.1285 (17)	
O8	0.2671 (4)	0.2615 (4)	0.5008 (3)	0.1301 (18)	
O9	0.4367 (5)	0.2216 (4)	0.5717 (3)	0.1331 (18)	
C1	0.6938 (4)	0.4314 (4)	0.3723 (3)	0.0514 (11)	
H1A	0.6469	0.4685	0.4103	0.062*	
C2	0.8274 (4)	0.4444 (4)	0.4061 (3)	0.0586 (13)	
H2A	0.8700	0.4908	0.4646	0.070*	
C3	0.8948 (4)	0.3864 (4)	0.3503 (3)	0.0625 (14)	
H3A	0.9836	0.3902	0.3715	0.075*	
C4	0.8300 (4)	0.3226 (4)	0.2628 (3)	0.0484 (11)	
H4	0.8751	0.2839	0.2242	0.058*	
C5	0.6979 (4)	0.3163 (3)	0.2325 (3)	0.0376 (10)	
C6	0.6255 (4)	0.2552 (3)	0.1382 (3)	0.0374 (10)	
C7	0.7001 (4)	0.2251 (4)	0.0665 (3)	0.0426 (10)	
C8	0.6898 (4)	0.1069 (4)	0.0231 (3)	0.0631 (13)	
H8	0.6357	0.0441	0.0385	0.076*	
C9	0.7599 (5)	0.0828 (5)	−0.0427 (4)	0.0842 (17)	
H9	0.7527	0.0034	−0.0720	0.101*	
C10	0.8401 (5)	0.1739 (6)	−0.0658 (4)	0.0833 (17)	
H10	0.8877	0.1564	−0.1101	0.100*	
C11	0.8501 (4)	0.2905 (5)	−0.0238 (3)	0.0689 (14)	
H11	0.9036	0.3525	−0.0404	0.083*	
C12	0.7816 (4)	0.3176 (4)	0.0431 (3)	0.0532 (12)	
H12	0.7901	0.3974	0.0723	0.064*	
C13	0.5020 (4)	0.5307 (4)	0.1411 (3)	0.0591 (13)	
H13	0.5472	0.4764	0.1168	0.071*	
C14	0.5201 (4)	0.6396 (4)	0.1186 (3)	0.0596 (13)	
H14	0.5765	0.6591	0.0806	0.071*	
C15	0.4529 (4)	0.7188 (4)	0.1534 (3)	0.0515 (12)	
H15	0.4614	0.7931	0.1385	0.062*	
C16	0.3725 (4)	0.6877 (3)	0.2106 (3)	0.0492 (11)	
H16	0.3262	0.7408	0.2349	0.059*	
C17	0.3608 (3)	0.5765 (3)	0.2320 (2)	0.0350 (9)	
C18	0.2810 (3)	0.5415 (3)	0.2966 (2)	0.0352 (9)	
C19	0.1797 (4)	0.6062 (3)	0.3191 (3)	0.0396 (10)	
C20	0.0747 (4)	0.6039 (4)	0.2504 (3)	0.0551 (12)	
H20	0.0691	0.5641	0.1903	0.066*	
C21	−0.0220 (5)	0.6609 (5)	0.2712 (4)	0.0768 (16)	
H21	−0.0921	0.6596	0.2246	0.092*	
C22	−0.0163 (6)	0.7188 (5)	0.3584 (5)	0.086 (2)	
H22	−0.0828	0.7553	0.3720	0.103*	
C23	0.0890 (6)	0.7224 (4)	0.4261 (4)	0.0761 (16)	
H23	0.0940	0.7627	0.4859	0.091*	
C24	0.1879 (4)	0.6674 (3)	0.4070 (3)	0.0553 (12)	
H24	0.2595	0.6719	0.4536	0.066*	
C25	0.5031 (4)	0.1182 (4)	0.3414 (3)	0.0510 (12)	
H25	0.5852	0.1662	0.3424	0.061*	
C26	0.5001 (4)	0.0196 (4)	0.3777 (3)	0.0570 (12)	
H26	0.5775	−0.0001	0.4014	0.068*	
C27	0.3793 (4)	−0.0487 (4)	0.3776 (3)	0.0508 (11)	
H27	0.3735	−0.1158	0.4024	0.061*	
C28	0.2668 (4)	−0.0187 (3)	0.3412 (3)	0.0498 (11)	
H28	0.1843	−0.0654	0.3405	0.060*	
C29	0.2771 (4)	0.0812 (3)	0.3057 (2)	0.0342 (9)	
C30	0.1588 (4)	0.1179 (3)	0.2634 (3)	0.0370 (9)	
C31	0.0258 (4)	0.0574 (3)	0.2733 (3)	0.0404 (10)	
C32	−0.0003 (4)	0.0635 (4)	0.3599 (3)	0.0518 (11)	
H32	0.0635	0.1083	0.4118	0.062*	
C33	−0.1227 (4)	0.0021 (4)	0.3680 (4)	0.0642 (14)	
H33	−0.1416	0.0074	0.4256	0.077*	
C34	−0.2156 (4)	−0.0661 (4)	0.2919 (4)	0.0690 (15)	
H34	−0.2964	−0.1086	0.2982	0.083*	
C35	−0.1901 (4)	−0.0721 (4)	0.2063 (4)	0.0726 (15)	
H35	−0.2538	−0.1182	0.1547	0.087*	
C36	−0.0694 (4)	−0.0095 (4)	0.1965 (3)	0.0597 (13)	
H36	−0.0527	−0.0125	0.1385	0.072*	

### Atomic displacement parameters (Å^2^)

**Table:**

	*U*^11^	*U*^22^	*U*^33^	*U*^12^	*U*^13^	*U*^23^
Cd1	0.03717 (17)	0.04326 (19)	0.0802 (3)	0.01777 (14)	0.02862 (17)	0.02601 (17)
N1	0.041 (2)	0.036 (2)	0.057 (3)	0.0085 (16)	0.0198 (19)	0.0055 (19)
N2	0.040 (2)	0.043 (2)	0.041 (2)	0.0079 (17)	0.0013 (18)	0.0117 (18)
N3	0.0376 (19)	0.043 (2)	0.054 (2)	0.0149 (16)	0.0173 (17)	0.0192 (18)
N4	0.051 (2)	0.046 (2)	0.038 (2)	0.0164 (17)	0.0197 (17)	0.0167 (18)
N5	0.0320 (19)	0.045 (2)	0.059 (2)	0.0132 (16)	0.0172 (17)	0.0218 (18)
N6	0.0314 (18)	0.051 (2)	0.048 (2)	0.0167 (17)	0.0053 (17)	0.0173 (19)
N7	0.034 (2)	0.087 (4)	0.074 (3)	0.015 (2)	0.007 (2)	0.010 (3)
N8	0.086 (4)	0.054 (3)	0.077 (4)	0.034 (3)	−0.007 (3)	0.003 (3)
O1	0.0427 (18)	0.064 (2)	0.052 (2)	0.0047 (17)	−0.0019 (16)	0.0074 (17)
O2	0.070 (2)	0.060 (2)	0.0440 (19)	0.0276 (16)	0.0248 (17)	0.0282 (16)
O3	0.0425 (17)	0.078 (2)	0.080 (2)	0.0250 (16)	0.0141 (16)	0.0456 (19)
O4	0.062 (2)	0.109 (3)	0.093 (3)	0.022 (2)	0.018 (2)	−0.016 (2)
O5	0.132 (4)	0.123 (3)	0.101 (3)	0.045 (3)	0.053 (3)	0.058 (3)
O6	0.078 (3)	0.104 (3)	0.129 (4)	0.041 (2)	0.043 (2)	0.054 (3)
O7	0.152 (4)	0.094 (3)	0.141 (4)	0.023 (3)	0.040 (3)	0.032 (3)
O8	0.074 (3)	0.107 (3)	0.177 (5)	0.026 (3)	−0.015 (3)	−0.005 (3)
O9	0.183 (5)	0.126 (4)	0.120 (4)	0.095 (4)	0.011 (3)	0.059 (3)
C1	0.054 (3)	0.049 (3)	0.047 (3)	0.009 (2)	0.017 (2)	0.002 (2)
C2	0.052 (3)	0.070 (3)	0.040 (3)	−0.005 (3)	0.005 (2)	0.007 (3)
C3	0.031 (2)	0.100 (4)	0.055 (3)	0.008 (3)	0.007 (2)	0.026 (3)
C4	0.037 (2)	0.075 (3)	0.036 (3)	0.019 (2)	0.009 (2)	0.012 (2)
C5	0.034 (2)	0.036 (2)	0.044 (3)	0.0074 (19)	0.015 (2)	0.010 (2)
C6	0.031 (2)	0.032 (2)	0.048 (3)	0.0077 (18)	0.009 (2)	0.008 (2)
C7	0.037 (2)	0.050 (3)	0.042 (3)	0.015 (2)	0.009 (2)	0.010 (2)
C8	0.075 (3)	0.054 (3)	0.068 (3)	0.024 (3)	0.029 (3)	0.012 (3)
C9	0.100 (4)	0.075 (4)	0.079 (4)	0.033 (3)	0.038 (4)	−0.005 (3)
C10	0.076 (4)	0.115 (5)	0.071 (4)	0.041 (4)	0.040 (3)	0.014 (4)
C11	0.053 (3)	0.105 (4)	0.056 (3)	0.016 (3)	0.020 (3)	0.033 (3)
C12	0.048 (3)	0.065 (3)	0.044 (3)	0.014 (2)	0.008 (2)	0.010 (2)
C13	0.057 (3)	0.054 (3)	0.085 (4)	0.020 (2)	0.043 (3)	0.031 (3)
C14	0.055 (3)	0.061 (3)	0.076 (4)	0.012 (2)	0.034 (3)	0.034 (3)
C15	0.055 (3)	0.041 (3)	0.058 (3)	0.002 (2)	0.010 (2)	0.022 (2)
C16	0.059 (3)	0.035 (2)	0.056 (3)	0.011 (2)	0.018 (2)	0.014 (2)
C17	0.029 (2)	0.037 (2)	0.036 (2)	0.0046 (18)	0.0019 (19)	0.008 (2)
C18	0.037 (2)	0.036 (2)	0.030 (2)	0.0083 (19)	0.0039 (19)	0.007 (2)
C19	0.049 (3)	0.039 (2)	0.039 (3)	0.018 (2)	0.015 (2)	0.015 (2)
C20	0.061 (3)	0.071 (3)	0.045 (3)	0.031 (3)	0.014 (2)	0.024 (3)
C21	0.063 (3)	0.089 (4)	0.109 (5)	0.045 (3)	0.032 (3)	0.058 (4)
C22	0.104 (5)	0.071 (4)	0.134 (6)	0.056 (4)	0.082 (5)	0.056 (4)
C23	0.117 (5)	0.056 (3)	0.083 (4)	0.042 (3)	0.062 (4)	0.026 (3)
C24	0.074 (3)	0.042 (3)	0.060 (3)	0.018 (2)	0.032 (3)	0.017 (2)
C25	0.030 (2)	0.054 (3)	0.073 (3)	0.010 (2)	0.008 (2)	0.029 (3)
C26	0.051 (3)	0.055 (3)	0.073 (4)	0.020 (2)	0.018 (3)	0.024 (3)
C27	0.060 (3)	0.041 (3)	0.056 (3)	0.018 (2)	0.014 (3)	0.019 (2)
C28	0.049 (3)	0.039 (3)	0.068 (3)	0.011 (2)	0.022 (2)	0.019 (2)
C29	0.038 (2)	0.035 (2)	0.031 (2)	0.0114 (19)	0.0088 (19)	0.0081 (19)
C30	0.035 (2)	0.036 (2)	0.039 (3)	0.0088 (19)	0.010 (2)	0.005 (2)
C31	0.032 (2)	0.042 (3)	0.049 (3)	0.010 (2)	0.012 (2)	0.012 (2)
C32	0.046 (3)	0.051 (3)	0.055 (3)	0.005 (2)	0.016 (2)	0.009 (2)
C33	0.054 (3)	0.063 (3)	0.082 (4)	0.011 (3)	0.040 (3)	0.012 (3)
C34	0.040 (3)	0.061 (3)	0.100 (5)	0.002 (3)	0.023 (3)	0.009 (3)
C35	0.039 (3)	0.082 (4)	0.075 (4)	−0.008 (3)	0.003 (3)	−0.001 (3)
C36	0.043 (3)	0.066 (3)	0.064 (3)	0.005 (2)	0.016 (3)	0.007 (3)

### Geometric parameters (Å, °)

**Table:**

Cd1—N3	2.320 (3)	C11—C12	1.380 (5)
Cd1—N1	2.337 (3)	C11—H11	0.9300
Cd1—N5	2.342 (3)	C12—H12	0.9300
Cd1—N6	2.376 (3)	C13—C14	1.368 (5)
Cd1—N4	2.380 (3)	C13—H13	0.9300
Cd1—N2	2.402 (3)	C14—C15	1.364 (5)
N1—C1	1.327 (5)	C14—H14	0.9300
N1—C5	1.353 (4)	C15—C16	1.374 (5)
N2—C6	1.279 (4)	C15—H15	0.9300
N2—O1	1.396 (4)	C16—C17	1.387 (5)
N3—C13	1.332 (4)	C16—H16	0.9300
N3—C17	1.341 (4)	C17—C18	1.474 (5)
N4—C18	1.285 (4)	C18—C19	1.486 (5)
N4—O2	1.378 (3)	C19—C24	1.372 (5)
N5—C25	1.340 (4)	C19—C20	1.382 (5)
N5—C29	1.349 (4)	C20—C21	1.382 (5)
N6—C30	1.269 (4)	C20—H20	0.9300
N6—O3	1.386 (3)	C21—C22	1.356 (7)
N7—O5	1.208 (5)	C21—H21	0.9300
N7—O4	1.227 (4)	C22—C23	1.370 (7)
N7—O6	1.252 (5)	C22—H22	0.9300
N8—O9	1.161 (5)	C23—C24	1.382 (6)
N8—O8	1.210 (5)	C23—H23	0.9300
N8—O7	1.234 (5)	C24—H24	0.9300
O1—H1	0.8200	C25—C26	1.371 (5)
O2—H2	0.8200	C25—H25	0.9300
O3—H3	0.8200	C26—C27	1.365 (5)
C1—C2	1.388 (5)	C26—H26	0.9300
C1—H1A	0.9300	C27—C28	1.369 (5)
C2—C3	1.373 (5)	C27—H27	0.9300
C2—H2A	0.9300	C28—C29	1.373 (5)
C3—C4	1.375 (5)	C28—H28	0.9300
C3—H3A	0.9300	C29—C30	1.490 (5)
C4—C5	1.380 (5)	C30—C31	1.491 (5)
C4—H4	0.9300	C31—C36	1.382 (5)
C5—C6	1.470 (5)	C31—C32	1.391 (5)
C6—C7	1.490 (5)	C32—C33	1.389 (5)
C7—C8	1.384 (5)	C32—H32	0.9300
C7—C12	1.387 (5)	C33—C34	1.368 (6)
C8—C9	1.373 (6)	C33—H33	0.9300
C8—H8	0.9300	C34—C35	1.374 (6)
C9—C10	1.366 (6)	C34—H34	0.9300
C9—H9	0.9300	C35—C36	1.387 (5)
C10—C11	1.362 (6)	C35—H35	0.9300
C10—H10	0.9300	C36—H36	0.9300
			
N3—Cd1—N1	86.23 (10)	C11—C12—C7	119.5 (4)
N3—Cd1—N5	172.80 (12)	C11—C12—H12	120.2
N1—Cd1—N5	88.33 (10)	C7—C12—H12	120.2
N3—Cd1—N6	117.48 (10)	N3—C13—C14	124.2 (4)
N1—Cd1—N6	156.28 (10)	N3—C13—H13	117.9
N5—Cd1—N6	68.00 (11)	C14—C13—H13	117.9
N3—Cd1—N4	69.06 (11)	C15—C14—C13	118.0 (4)
N1—Cd1—N4	110.42 (11)	C15—C14—H14	121.0
N5—Cd1—N4	108.62 (11)	C13—C14—H14	121.0
N6—Cd1—N4	80.07 (11)	C14—C15—C16	119.2 (4)
N3—Cd1—N2	89.14 (10)	C14—C15—H15	120.4
N1—Cd1—N2	68.22 (12)	C16—C15—H15	120.4
N5—Cd1—N2	93.23 (10)	C15—C16—C17	119.7 (4)
N6—Cd1—N2	110.04 (11)	C15—C16—H16	120.2
N4—Cd1—N2	158.13 (10)	C17—C16—H16	120.2
C1—N1—C5	118.6 (3)	N3—C17—C16	121.0 (3)
C1—N1—Cd1	122.8 (3)	N3—C17—C18	117.3 (3)
C5—N1—Cd1	117.9 (3)	C16—C17—C18	121.6 (3)
C6—N2—O1	113.9 (3)	N4—C18—C17	115.1 (3)
C6—N2—Cd1	119.3 (3)	N4—C18—C19	124.6 (3)
O1—N2—Cd1	126.3 (2)	C17—C18—C19	120.2 (3)
C13—N3—C17	117.8 (3)	C24—C19—C20	119.3 (4)
C13—N3—Cd1	125.2 (3)	C24—C19—C18	121.3 (4)
C17—N3—Cd1	116.9 (2)	C20—C19—C18	119.4 (4)
C18—N4—O2	114.0 (3)	C21—C20—C19	119.8 (4)
C18—N4—Cd1	115.8 (2)	C21—C20—H20	120.1
O2—N4—Cd1	123.5 (2)	C19—C20—H20	120.1
C25—N5—C29	117.2 (3)	C22—C21—C20	121.1 (5)
C25—N5—Cd1	124.3 (3)	C22—C21—H21	119.5
C29—N5—Cd1	118.5 (2)	C20—C21—H21	119.5
C30—N6—O3	114.2 (3)	C21—C22—C23	118.9 (5)
C30—N6—Cd1	119.8 (3)	C21—C22—H22	120.6
O3—N6—Cd1	123.4 (2)	C23—C22—H22	120.6
O5—N7—O4	122.9 (5)	C22—C23—C24	121.2 (5)
O5—N7—O6	120.6 (5)	C22—C23—H23	119.4
O4—N7—O6	116.5 (5)	C24—C23—H23	119.4
O9—N8—O8	126.6 (6)	C19—C24—C23	119.6 (5)
O9—N8—O7	120.3 (6)	C19—C24—H24	120.2
O8—N8—O7	112.9 (5)	C23—C24—H24	120.2
N2—O1—H1	109.5	N5—C25—C26	124.1 (4)
N4—O2—H2	109.5	N5—C25—H25	118.0
N6—O3—H3	109.5	C26—C25—H25	118.0
N1—C1—C2	123.3 (4)	C27—C26—C25	117.6 (4)
N1—C1—H1A	118.4	C27—C26—H26	121.2
C2—C1—H1A	118.4	C25—C26—H26	121.2
C3—C2—C1	117.8 (4)	C26—C27—C28	120.1 (4)
C3—C2—H2A	121.1	C26—C27—H27	120.0
C1—C2—H2A	121.1	C28—C27—H27	120.0
C2—C3—C4	119.5 (4)	C27—C28—C29	119.3 (4)
C2—C3—H3A	120.2	C27—C28—H28	120.4
C4—C3—H3A	120.2	C29—C28—H28	120.4
C3—C4—C5	119.7 (4)	N5—C29—C28	121.8 (3)
C3—C4—H4	120.1	N5—C29—C30	116.0 (3)
C5—C4—H4	120.1	C28—C29—C30	122.2 (3)
N1—C5—C4	121.0 (4)	N6—C30—C29	115.6 (3)
N1—C5—C6	116.9 (3)	N6—C30—C31	125.1 (3)
C4—C5—C6	122.0 (3)	C29—C30—C31	119.2 (3)
N2—C6—C5	116.2 (3)	C36—C31—C32	120.0 (4)
N2—C6—C7	123.9 (4)	C36—C31—C30	119.9 (4)
C5—C6—C7	119.6 (3)	C32—C31—C30	120.0 (4)
C8—C7—C12	119.5 (4)	C33—C32—C31	119.3 (4)
C8—C7—C6	121.3 (4)	C33—C32—H32	120.4
C12—C7—C6	119.2 (4)	C31—C32—H32	120.4
C9—C8—C7	119.6 (4)	C34—C33—C32	120.5 (4)
C9—C8—H8	120.2	C34—C33—H33	119.8
C7—C8—H8	120.2	C32—C33—H33	119.8
C10—C9—C8	120.9 (5)	C33—C34—C35	120.3 (4)
C10—C9—H9	119.5	C33—C34—H34	119.8
C8—C9—H9	119.5	C35—C34—H34	119.8
C11—C10—C9	119.8 (5)	C34—C35—C36	120.1 (5)
C11—C10—H10	120.1	C34—C35—H35	119.9
C9—C10—H10	120.1	C36—C35—H35	119.9
C10—C11—C12	120.6 (5)	C31—C36—C35	119.8 (4)
C10—C11—H11	119.7	C31—C36—H36	120.1
C12—C11—H11	119.7	C35—C36—H36	120.1
			
N3—Cd1—N1—C1	90.4 (3)	N2—C6—C7—C8	−69.3 (5)
N5—Cd1—N1—C1	−84.8 (3)	C5—C6—C7—C8	116.4 (4)
N6—Cd1—N1—C1	−88.5 (4)	N2—C6—C7—C12	110.8 (5)
N4—Cd1—N1—C1	24.4 (3)	C5—C6—C7—C12	−63.5 (5)
N2—Cd1—N1—C1	−179.0 (3)	C12—C7—C8—C9	−0.3 (7)
N3—Cd1—N1—C5	−99.7 (3)	C6—C7—C8—C9	179.8 (4)
N5—Cd1—N1—C5	85.0 (3)	C7—C8—C9—C10	0.2 (8)
N6—Cd1—N1—C5	81.4 (4)	C8—C9—C10—C11	−0.6 (9)
N4—Cd1—N1—C5	−165.7 (2)	C9—C10—C11—C12	1.1 (8)
N2—Cd1—N1—C5	−9.1 (2)	C10—C11—C12—C7	−1.1 (7)
N3—Cd1—N2—C6	89.4 (3)	C8—C7—C12—C11	0.7 (6)
N1—Cd1—N2—C6	3.1 (3)	C6—C7—C12—C11	−179.4 (4)
N5—Cd1—N2—C6	−83.8 (3)	C17—N3—C13—C14	−1.2 (7)
N6—Cd1—N2—C6	−151.5 (3)	Cd1—N3—C13—C14	−176.6 (3)
N4—Cd1—N2—C6	93.9 (4)	N3—C13—C14—C15	−0.7 (7)
N3—Cd1—N2—O1	−81.9 (3)	C13—C14—C15—C16	1.3 (7)
N1—Cd1—N2—O1	−168.2 (3)	C14—C15—C16—C17	−0.1 (6)
N5—Cd1—N2—O1	104.9 (3)	C13—N3—C17—C16	2.4 (6)
N6—Cd1—N2—O1	37.2 (3)	Cd1—N3—C17—C16	178.2 (3)
N4—Cd1—N2—O1	−77.4 (4)	C13—N3—C17—C18	−176.4 (4)
N1—Cd1—N3—C13	52.5 (3)	Cd1—N3—C17—C18	−0.6 (4)
N6—Cd1—N3—C13	−128.0 (3)	C15—C16—C17—N3	−1.8 (6)
N4—Cd1—N3—C13	166.1 (4)	C15—C16—C17—C18	177.0 (4)
N2—Cd1—N3—C13	−15.7 (3)	O2—N4—C18—C17	179.7 (3)
N1—Cd1—N3—C17	−122.9 (3)	Cd1—N4—C18—C17	−27.8 (4)
N6—Cd1—N3—C17	56.6 (3)	O2—N4—C18—C19	1.9 (5)
N4—Cd1—N3—C17	−9.3 (3)	Cd1—N4—C18—C19	154.4 (3)
N2—Cd1—N3—C17	168.9 (3)	N3—C17—C18—N4	19.4 (5)
N3—Cd1—N4—C18	20.4 (3)	C16—C17—C18—N4	−159.4 (4)
N1—Cd1—N4—C18	97.8 (3)	N3—C17—C18—C19	−162.7 (3)
N5—Cd1—N4—C18	−166.9 (3)	C16—C17—C18—C19	18.5 (6)
N6—Cd1—N4—C18	−104.3 (3)	N4—C18—C19—C24	58.9 (6)
N2—Cd1—N4—C18	15.6 (5)	C17—C18—C19—C24	−118.8 (4)
N3—Cd1—N4—O2	170.0 (3)	N4—C18—C19—C20	−120.5 (4)
N1—Cd1—N4—O2	−112.6 (3)	C17—C18—C19—C20	61.8 (5)
N5—Cd1—N4—O2	−17.3 (3)	C24—C19—C20—C21	−1.3 (6)
N6—Cd1—N4—O2	45.3 (3)	C18—C19—C20—C21	178.1 (4)
N2—Cd1—N4—O2	165.2 (3)	C19—C20—C21—C22	−0.5 (7)
N1—Cd1—N5—C25	−4.0 (3)	C20—C21—C22—C23	1.5 (8)
N6—Cd1—N5—C25	174.4 (3)	C21—C22—C23—C24	−0.7 (8)
N4—Cd1—N5—C25	−115.1 (3)	C20—C19—C24—C23	2.1 (6)
N2—Cd1—N5—C25	64.0 (3)	C18—C19—C24—C23	−177.3 (4)
N1—Cd1—N5—C29	174.6 (3)	C22—C23—C24—C19	−1.1 (7)
N6—Cd1—N5—C29	−7.0 (3)	C29—N5—C25—C26	1.7 (6)
N4—Cd1—N5—C29	63.5 (3)	Cd1—N5—C25—C26	−179.7 (3)
N2—Cd1—N5—C29	−117.4 (3)	N5—C25—C26—C27	−1.4 (7)
N3—Cd1—N6—C30	−161.7 (3)	C25—C26—C27—C28	0.8 (6)
N1—Cd1—N6—C30	17.1 (5)	C26—C27—C28—C29	−0.6 (6)
N5—Cd1—N6—C30	13.1 (3)	C25—N5—C29—C28	−1.4 (5)
N4—Cd1—N6—C30	−101.7 (3)	Cd1—N5—C29—C28	179.9 (3)
N2—Cd1—N6—C30	98.4 (3)	C25—N5—C29—C30	−179.4 (3)
N3—Cd1—N6—O3	−1.1 (3)	Cd1—N5—C29—C30	1.9 (4)
N1—Cd1—N6—O3	177.7 (3)	C27—C28—C29—N5	1.0 (6)
N5—Cd1—N6—O3	173.7 (3)	C27—C28—C29—C30	178.8 (4)
N4—Cd1—N6—O3	58.9 (3)	O3—N6—C30—C29	−179.0 (3)
N2—Cd1—N6—O3	−101.0 (3)	Cd1—N6—C30—C29	−16.7 (4)
C5—N1—C1—C2	−1.3 (6)	O3—N6—C30—C31	2.5 (5)
Cd1—N1—C1—C2	168.5 (3)	Cd1—N6—C30—C31	164.8 (3)
N1—C1—C2—C3	−1.7 (6)	N5—C29—C30—N6	9.7 (5)
C1—C2—C3—C4	2.7 (6)	C28—C29—C30—N6	−168.2 (4)
C2—C3—C4—C5	−0.8 (6)	N5—C29—C30—C31	−171.7 (3)
C1—N1—C5—C4	3.4 (5)	C28—C29—C30—C31	10.4 (5)
Cd1—N1—C5—C4	−167.0 (3)	N6—C30—C31—C36	61.6 (5)
C1—N1—C5—C6	−175.6 (3)	C29—C30—C31—C36	−116.9 (4)
Cd1—N1—C5—C6	14.1 (4)	N6—C30—C31—C32	−121.4 (4)
C3—C4—C5—N1	−2.3 (6)	C29—C30—C31—C32	60.1 (5)
C3—C4—C5—C6	176.5 (4)	C36—C31—C32—C33	−0.1 (6)
O1—N2—C6—C5	175.1 (3)	C30—C31—C32—C33	−177.1 (4)
Cd1—N2—C6—C5	2.7 (4)	C31—C32—C33—C34	1.6 (7)
O1—N2—C6—C7	0.6 (5)	C32—C33—C34—C35	−1.7 (7)
Cd1—N2—C6—C7	−171.7 (3)	C33—C34—C35—C36	0.4 (8)
N1—C5—C6—N2	−11.1 (5)	C32—C31—C36—C35	−1.2 (6)
C4—C5—C6—N2	170.0 (4)	C30—C31—C36—C35	175.8 (4)
N1—C5—C6—C7	163.6 (3)	C34—C35—C36—C31	1.0 (7)
C4—C5—C6—C7	−15.3 (5)		

### Hydrogen-bond geometry (Å, °)

**Table:**

*D*—H···*A*	*D*—H	H···*A*	*D*···*A*	*D*—H···*A*
C32—H32···O8	0.93	2.48	3.355 (6)	157
C27—H27···O9^i^	0.93	2.58	3.191 (6)	123
C14—H14···O5^ii^	0.93	2.49	3.261 (6)	141
C4—H4···O3^iii^	0.93	2.42	3.285 (5)	155
C3—H3A···O2^iii^	0.93	2.56	3.461 (5)	164
O3—H3···O6	0.82	2.34	2.892 (4)	126
O3—H3···O4	0.82	2.00	2.820 (5)	176
O2—H2···O8	0.82	2.04	2.800 (5)	154
O2—H2···O7	0.82	2.21	2.939 (6)	149
O1—H1···O6	0.82	1.81	2.585 (4)	158

Symmetry codes: (i) −*x*+1, −*y*, −*z*+1; (ii) −*x*+1, −*y*+1, −*z*; (iii) *x*+1, *y*, *z*.
